# Integrated Adsorption–Photodegradation of Organic Pollutants by Carbon Xerogel/Titania Composites

**DOI:** 10.3390/molecules27238483

**Published:** 2022-12-02

**Authors:** Anam Safri, Ashleigh Jane Fletcher, Ramsha Safri, Hifza Rasheed

**Affiliations:** 1 Department of Chemical and Process Engineering, University of Strathclyde, Glasgow G1 1XJ, UK; 2 College of Medicine and Surgery, The Shaheed Zulfiqar Ali Bhutto Medical University Islamabad, Islamabad 44000, Pakistan; 3 Pakistan Council of Research in Water Resources (PCRWR), Islamabad 44000, Pakistan

**Keywords:** carbon xerogel, TiO_2_ photocatalysis, adsorption–photodegradation, dye degradation, adsorption isotherm, adsorption kinetics, microbial degradation

## Abstract

Recent studies on the removal of pollutants via adsorption include the use of carbon-based adsorbents, due to their high porosity and large surface area; however, such materials lack photoactive properties. This study evaluates the synergistic effect of integrated mesoporous carbon xerogel (derived from resorcinol formaldehyde) and titanium dioxide (TiO_2_) for combined adsorption and photodegradation application. The complex formed between carbon xerogel and TiO_2_ phase was investigated through FTIR, proving the presence of a Ti-O–C chemical linkage. The physicochemical properties of the synthesised adsorbent–photocatalyst were probed using FESEM, BET analysis and UV–Vis analysis. The kinetics, equilibrium adsorption, effect of pH, and effect of adsorbent dosage were investigated. The expansion of the absorbance range to the visible range was verified, and the corresponding band gap evaluated. These properties enabled a visible light response when the system was exposed to visible light post adsorption. Hence, an assistive adsorption–photodegradation phenomenon was successfully executed. The adsorption performance exhibited 85% dye degradation which improved to 99% following photodegradation. Further experiments showed the reduction of microorganisms under visible light, where no microbial colonies were observed after treatment, indicating the potential application of these composite materials.

## 1. Introduction

Amongst several conventional water treatment techniques, adsorption is recognised as one of the most simple, reliable, and effective methods. New techniques use nano adsorbents, of which one of the main groups for wastewater treatment are carbon-based adsorbents [1]. Generally, carbon is preferred, due to its high effectiveness, abundant availability, and low cost. However, adsorption using basic carbon materials is restricted by slow kinetics, while advanced adsorbents, for example, zeolites or metal-based nano-adsorbents are expensive, and experience a loss of adsorption sites during the desorption process, resulting in pollution during the application process and requiring high energy for their regeneration [2]. 

Photodegradation techniques use light irradiance for the activation of a photocatalyst, upon which they generate charge carriers which transfer to the surface and undergo a series of chemical reactions to generate reactive oxide species or hydroxyl radicals. These species target the pollutant surface and chemically decompose it into harmless substances [3]. Titanium dioxide (TiO_2_) is widely studied in this regard, due to its non-toxicity, cost efficiency and ability to oxidise organic pollutants and eventually convert them to carbon dioxide and water [4]. The major limitations encountered by TiO_2_, which restricts the process efficiency, include agglomeration, wide band gap and rapid recombination of photogenerated charge carriers. Nevertheless, the adsorption capacity on the surface of TiO_2_ is poor; therefore, the degradation process is restricted. The use of carbon as a support matrix can not only enhance the adsorption capacity, but also solve the problem of charge recombination [5]. Additionally, chemical functionalisation can modify the bandgap of TiO_2_; hence, enhanced performance of an integrated system under visible light is possible.

As a consequence of the likelihood of improved performance through composite development, several studies have reported on the functional crosslinking of carbon and TiO_2_ through a wide range of synthesis procedures [6]. Sol–gel is commonly employed, as the synthesis process can be controlled to achieve strong chemical bonding and dispersibility of TiO_2_ nanoparticles. Other methods are expensive, time-consuming and difficult to implement, due to stringent control of factors [7]. With regard to material properties, studies related to chemical interactions between carbon and TiO_2_ are limited. Not many studies report on the complex formation between functional moieties of carbon and TiO_2_, which is theoretically responsible for the modification of the electronic structure and thus enables visible light response [8]. In this study, we have integrated TiO_2_ in a nano mesoporous carbon xerogel (CX) to obtain a visible light-activated adsorbent–photocatalyst. CX was derived from the polycondensation of resorcinol and formaldehyde in the presence of a base catalyst. Resorcinol formaldehyde gels have been utilised for several applications including thermal insulation [9], electrical conductivity [10], adsorption, and gas storage [11]. However, less attention has been paid to studying their application in the water treatment sector, particularly for visible light photocatalysis. Our reasons for choosing resorcinol–formaldehyde as a carbon source to produce CX were (i) it is composed of a unique structure (aromatic rings and OH groups), which can bind with TiO_2_ to produce new electronic interactions between CX and TiO_2_ and activate visible light response; and (ii) the materials are generally highly mesoporous structures, possessing large surface areas for effective adsorption of pollutants. Within the nanostructure, homogenously distributed TiO_2_ forms a heterojunction with the carbon substrate, due to the chemical interaction between CX and TiO_2_, resulting in complex formation at the interface of the material. Remarkably, such structures can absorb visible light and exhibit photoactivity under visible light irradiation.

This indicates that integrated CX and TiO_2_ may be an effective adsorbent–photocatalyst to target a wide range of pollutants in contaminated water, including dyes and microbes. Thus, a combined photocatalytic adsorbent, CXTi, was synthesised to exploit the synergistic effect of both adsorption and photocatalysis. The ratios of CX and TiO_2_ were carefully selected to optimise the physicochemical and optical properties imperative to maximise adsorbent–adsorbate interactions and utilisation of a wide fraction of the electromagnetic spectrum. The synthesised CXTi was tested against adsorption and photodegradation of methylene blue (MB) from aqueous solutions, and the acquired data analysed using kinetic and isothermal analysis. Additionally, antimicrobial tests were performed against the reduction of an indicator microorganism, i.e., total faecal coliform.

## 2. Results and Discussion

### 2.1. Structural Properties of CXTi

**Figure 1:**
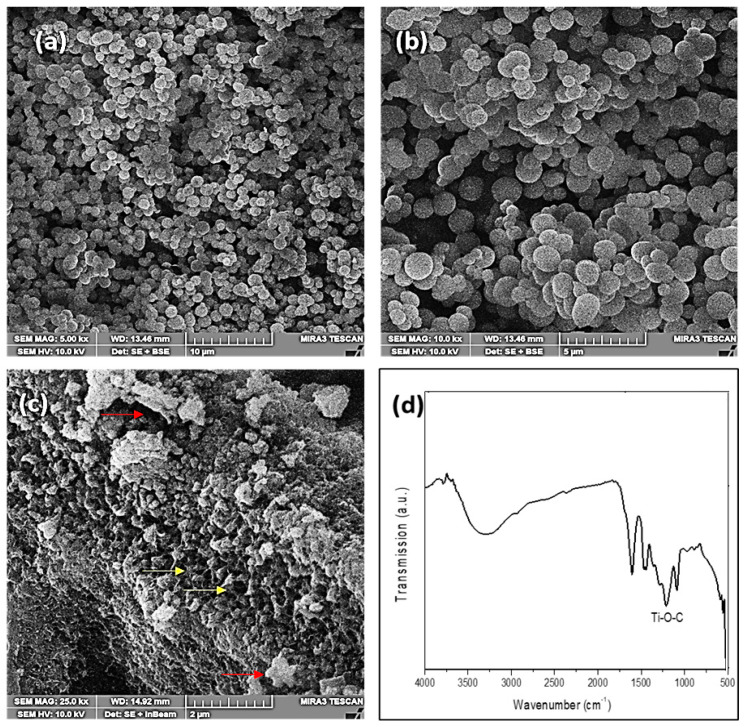
(**a**–**c**) FESEM micrographs (contrast altered) at different magnifications and (**d**) FTIR spectrum of CXTi synthesised in this study.

Figure 1a–c show micrographs of synthesised CXTi at different magnifications, which confirm that the carbons phase developed as a spherical form, as previously reported for carbons derived from the polycondensation and subsequent pyrolysis of resorcinol and formaldehyde gels [12]. The micrographs reveal uniform microclusters of interconnected microspheres with diameters in the range 1.2–1.5 μm. The surface heterogeneity/roughness observed in a section of the micrograph in Figure 1c, marked by red arrows, denotes carbon xerogel spheres engulfed by TiO_2_ nanoparticles. The yellow arrows show the porosity in the CXTi structure. The chemical bonding between carbon and TiO_2_ was confirmed by the recorded FTIR spectrum, shown in Figure 1d, where characteristic peaks of a typical resorcinol–formaldehyde-derived carbon were observed, including signals from C-H, C=C, C-O-C of aromatic rings and methylene bridges [13]. The broad peak at 3300 cm^−1^ is associated with OH of the phenolic groups. The vibrations in the range 2000–1700 are ascribed to C-H bending of aromatic moieties. The aromatic ether bridges formed during the polycondensation of resorcinol (R) and formaldehyde (F) result in the absorption bands observed at 1605 and 1473 cm^−1^. These observations are in agreement with those reported by Awadallah et al., for the mesoporous RF xerogels [14]. The chemical bonding between the carbon xerogel and TiO_2_ was confirmed by Ti-O-C functionalities in the range 1200–1000 cm^−1^ [15], indicating that the surface moieties of carbon xerogel support the attachment of TiO_2_. This heterojunction has been reported as a charge–transfer complex, which promotes electronic interactions and enhances the visible light response of the integrated carbon and TiO_2_ material [16,17]. Furthermore, signals below 1000 cm^−1^ are attributed to functional groups of titanium ethoxide and Ti-O-Ti linkages [18].

**Figure 2:**
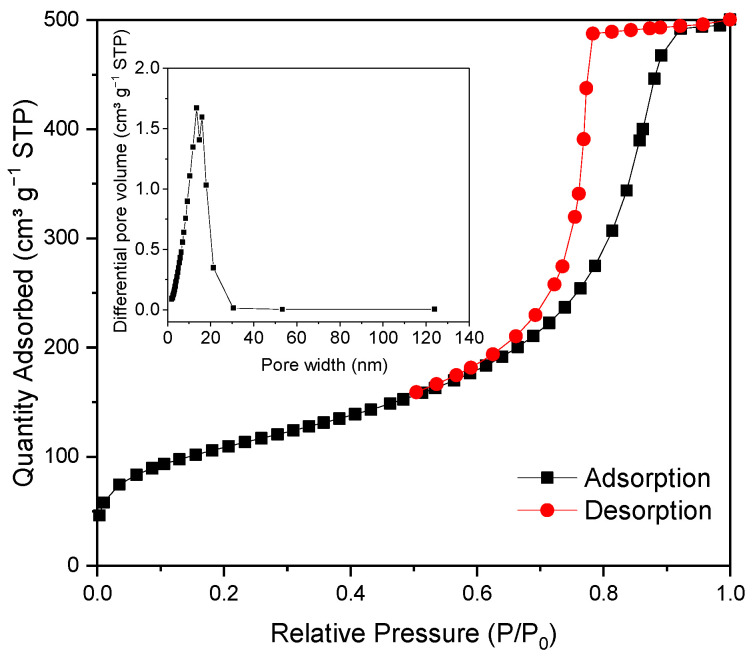
Nitrogen adsorption/desorption isotherm of CXTi synthesised in this study.

The surface area and porosity of synthesised CXTi were obtained using nitrogen sorption isotherms and subsequent BJH analysis to determine the pore size distributions [19], as shown in Figure 2. The isotherm shows that the characteristics of a typical mesoporous carbon xerogel derived from resorcinol–formaldehyde are retained [12]. The BET surface area and pore volume were calculated to be 384 m^2^ g^−1^ and 0.8 cm^3^ g^−1^, respectively. A narrow pore size distribution was observed, and the average pore width was calculated to be ~9 nm, as represented in the inset of Figure 2. It is evident that the shape of the isotherm is Type IV with a distinct hysteresis loop of Type H1 [20]. These findings suggest the presence of ordered mesopores with a uniform cylindrical shaped, open-ended three-dimensional pore network [20]. The calculated mesoporosity in the structure was 93%. These textural properties, comprising high surface area and well-developed mesoporous structure, are expected to facilitate the adsorption process and enhance the photocatalytic activity. 

**Figure 3:**
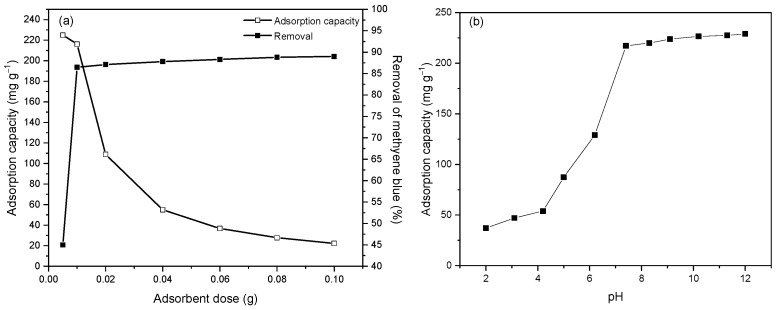
Effect of (**a**) adsorbent dosage and (**b**) pH on the adsorption capacity of methylene blue using CXTi synthesised in this study (T = 23 °C, adsorbent dose = 0.01 g, C_0_ = 100 mg L^−1^).

High adsorbent loading appears to reduce adsorption performance, while low adsorbent loading results in fewer pollutant molecules interacting with the active sites on the adsorbent; hence, the amount of adsorbent used is crucial in determining the ultimate adsorption performance. The study of the effect of dosage rate of CXTi on adsorption performance was performed at an initial concentration of 100 mg L^−1^ of MB (Figure 3a). It was observed that the amount of MB adsorbed increased rapidly with increasing adsorbent dose, from 0.005 to 0.01 g, tested against 25 mL of MB solution. This is attributed to the large surface area, mesoporous character and availability of vacant adsorptive sites associated with the CXTi sorbent. Further increase in adsorbent dose, up to 0.10 g, showed no significant increase in removal of MB. At this point, the concentration of adsorbate on the surface of the adsorbent, and the adsorptive in solution, reach equilibrium. Accordingly, 0.01 g was chosen as the optimal amount of adsorbent per 25 mL to conduct the following adsorption experiments. 

Additionally, for optimum adsorption performance, it is important to determine a suitable operating pH, since this affects the surface charge and ionisation of pollutant molecules, while unfavourable adsorption, dissolution and decomposition lead to low adsorption uptakes [21]. Figure 3b shows the effect of pH on the adsorption of MB by CXTi, as a function of pH value. The adsorption capacity of MB at acidic pH (≤6) was low, which may be due to the positively charged sample surface repelling the cationic MB dye molecules. At higher pH values, i.e., more basic, in the range of 8–12, the interface of CXTi is negatively charged, which means it can favourably adsorb the cationic MB molecule; hence, enhanced adsorption capacity is observed. Therefore, a pH of ~7.2 was maintained for all remaining adsorption experiments.

### 2.2. Adsorption Kinetics 

**Figure 4:**
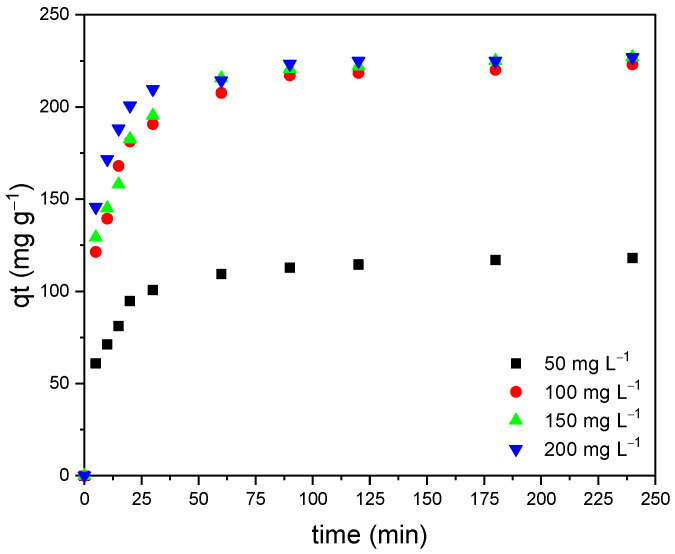
Effect of contact time (0–240 min) on MB adsorption on CXTi synthesised in this study, at initial concentrations of 50, 100, 150 and 200 mg L^−1^. Note: Error bars are negligibly small, hence the overlap of the limits.

Figure 4 shows the kinetic data obtained for MB adsorption on CXTi at different initial concentrations (50–200 mg L^−1^) and contact times (0–240 min). It was observed that the adsorption capacity increases with increase in initial concentration, as time elapsed. This is attributed to increased frequency of collisions between MB molecules and the sample surface, which overcomes the resistance to mass transfer between the adsorbate and the adsorbent, and results in the immediate occupancy of available active sites [22]. Rapid attainment of equilibrium is attributed to the highly porous nature of the sample with abundant active sites. In this case, π-π interactions between the MB dye molecules and the aromatic groups of the carbon xerogel, along with electrostatic interactions between the MB dye molecules and the hydroxyl groups of TiO_2_, are predominant binding strengths between the adsorbent and adsorbate. The adsorption process slows after this initial phase and eventually attains equilibrium at 90 min, as the mass transfer rate slows down due to saturation of the active sites, thus making it difficult for the MB molecules to further adsorb on the sample surface. In addition to this, higher initial concentrations of the adsorptive may result in aggregation of MB or charge repulsion of MB dye species, thereby decelerating the adsorption process [23]; thus, it takes longer for the adsorptive to diffuse deeper into the pores of the adsorbent.

To gain a better understanding of the kinetic and diffusion behaviour of the adsorptive–adsorbent system, the experimental data were analysed by models including Pseudo First Order (PFO), Pseudo Second Order (PSO), Elovich, and Intra-Particle Diffusion (IPD) [24]. The PFO model has frequently been used to describe kinetic processes under non-equilibrium conditions. PFO assumes that the rate of adsorption is proportional to the driving force, i.e., the difference between equilibrium concentration and solid phase concentration. The integrated form of the PFO model is given in Equation (1):

(1) $$q_{t\;=}{\;q}_{e}\left(1-e^{k_{1}t}\right)$$

By contrast, the PSO equation assumes that the overall adsorption rate is proportional to the square of the driving force. The integrated form of PSO model is given in Equation (2):

(2) $$q_{t}=\frac{k_{2}{\mathrm{tq}}_{e}^{2}}{1+k_{2}{\mathrm{tq}}_{e}}$$

In both Equations (1) and (2), q_t_ (mg g^−1^) and q_e_ (mg g^−1^) are the concentrations of MB dye molecules at time t and at equilibrium, respectively. k_1_ (g mg^−1^ min^−1^) and k_2_ (g^−1^ mg min^−1^) are the PFO and PSO rate constants, respectively.

**Figure 5:**
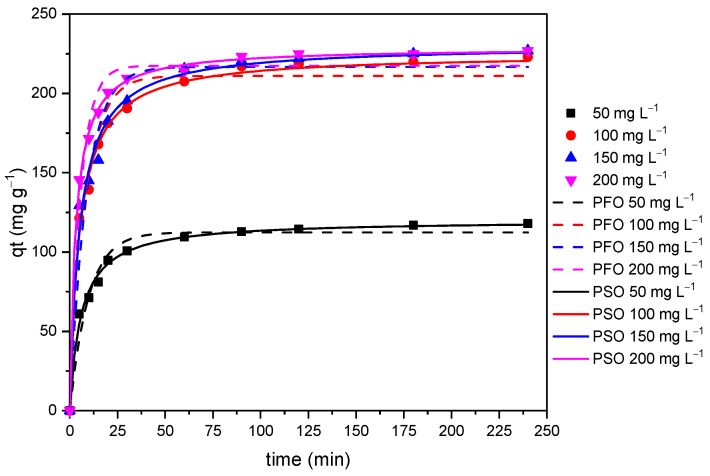
Pseudo first order and pseudo second order model fittings to experimental data for adsorption of MB on CXTi synthesised in this study, at initial concentrations of 50, 100, 150 and 200 mg L^−1^ (T = 23 °C, dose = 0.01 g mL^−1^).

Figure 5 shows the experimentally determined adsorption (q_exp_) data fitted to the PFO and PSO models. The evaluated parameters, including q_exp_, q_t_ (adsorption calculated at given time), first order rate constant k_1_, second order rate constant k_2_, and regression coefficient, are presented in Table 1. It was observed that PFO is incompatible with the experimental data, as the regression coefficient deviates from 1 throughout the concentration range; hence, physisorption is less likely to be the primary adsorption phenomenon.

By contrast, the regression coefficient values obtained for PSO are much closer to 1, indicating a better fit to the experimental data, and the calculated adsorption capacities through PSO equation closely match with the experimentally determined adsorption capacities (q_exp_), as shown in Table 1. Hence, the system can be best interpreted by the PSO model, and thus it can be concluded that the interaction between the surface of CXTi and MB is chemical in nature, and MB is mainly removed by a chemisorption phenomenon [25]. 

**Figure 6:**
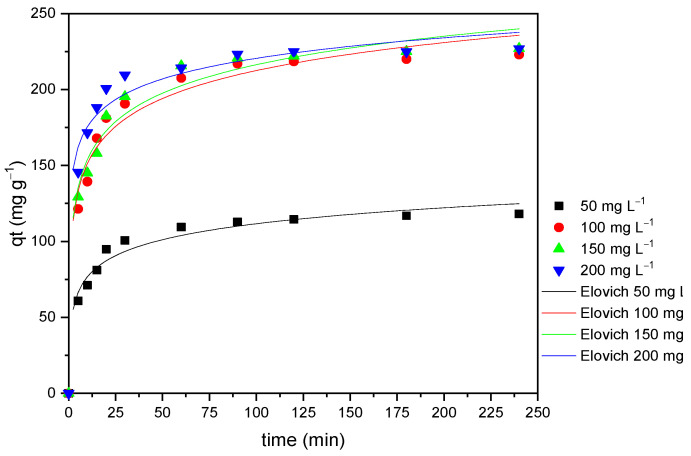
Elovich model fitting to experimental data for adsorption of MB on CXTi synthesised in this study, at initial concentrations of 50, 100, 150 and 200 mg L^−1^ (T = 23 °C, dose = 0.01 g mL^−1^).

**Figure 7:**
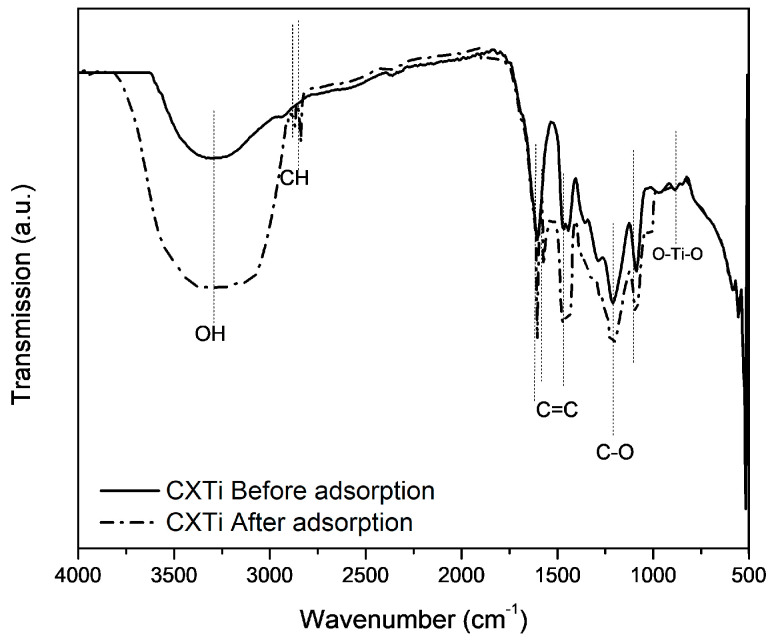
FTIR spectra of CXTi recorded before and after the adsorption treatment.

To further verify the chemisorption phenomenon of adsorption, the kinetic data was fitted to the Elovich equation, as shown in Equation (3) [24].

(3) $$q_{t}=\frac{1}{B}\ln\left(\mathrm{AB}\right)+\frac{1}{B}\ln\left(t\right)$$

where A (mg g^−1^ min^−1^) and B (g mg^−1^) are the initial rate constant of adsorption and desorption, respectively. This equation has been applied to wastewater treatment systems and is useful in describing chemisorption processes, which involve valence forces through sharing or exchange between the adsorbate and the adsorbent. The equation also signifies that the removal efficiency decreases with time because of the coverage of active sites [24]. The fit of the experimental data to the Elovich model is shown in Figure 6, and the obtained fits are good (R^2^ > 0.97), therefore verifying chemisorption within the adsorption process [26]. In addition, FTIR spectra of the adsorbent were recorded after adsorption treatment to confirm that the functional groups on CXTi are responsible for the observed adsorption activity. As shown in Figure 7, the change in spectra before and after adsorption of MB is evident, where the peaks associated with C-H, C=C and C-O-C vibrations appeared intense in comparison with CXTi before adsorption. Thus, it can be concluded that the adsorption of MB onto CXTi occurred predominantly via π–π interactions due to the aromatic rings of carbon xerogel [27].

To further understand the internal diffusion mechanism within the adsorbate–adsorbent system, the IPD model was applied to the experimental data. The IPD model is commonly applied to study the rate limiting step during the adsorption process, which is defined by either mass transfer or the diffusion of adsorbate and pore diffusion. IPD is studied by fitting the data to Equation (4) [24].

(4) $$q_{t}=k_{\mathrm{ip}}t^{0.5}+C$$

k_ip_ is the rate constant (mg^−1^ g min^0.5^) and the intercept C is the boundary layer thickness. The value of C defines the boundary layer effect. A plot of qt vs. t^0.5^ gives a linear function; if the line passes through the origin, IP diffusion controls the adsorption process. If the line does not pass through origin and shows multiple linear segments, these segments correspond to different mechanisms that control the adsorption process [24]. 

**Figure 8:**
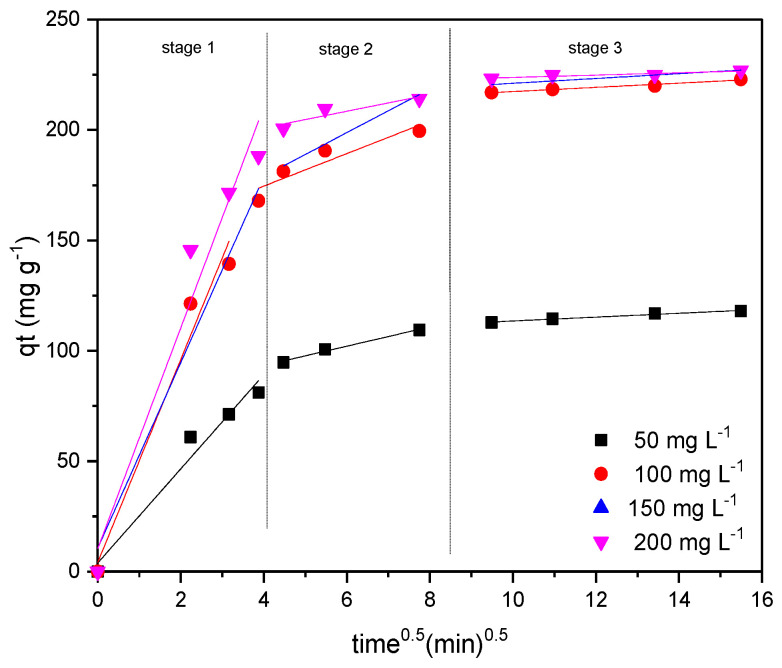
The intra-particle diffusion model linearly fitted to experimental data for adsorption of MB onto CXTi synthesised in this study, at initial concentrations of 50, 100, 150 and 200 mg L^−1^ (T = 23 °C, dose = 0.01 g mL^−1^).

Figure 8 shows the plot of MB adsorption onto CXTi fitted to the IPD equation plotted against t^0.5^. The multistep adsorption process is evident through the multi-linearity of the data (marked as stages 1, 2 and 3), which indicates that intra-particle diffusion was not rate limiting, and other adsorption mechanisms were also involved [25]. This means that the adsorption process, stage 1, began with rapid diffusion of MB from the bulk phase to the external surface of the sample, adsorbing swiftly due to the immediate availability of a large proportion of adsorption sites. The second stage was slower due to the boundary layer effect causing slow diffusion of the adsorptive into the porous structure of the sample. The third stage suggests an equilibrium stage, where intra-particle diffusion starts to slow down due to low adsorptive concentration or saturation of the active sites, preventing surface reactions from occurring [24,25]. The calculated parameters obtained by piecewise fitting are shown in Supplementary Materials, Table S1.

### 2.3. Adsorption Isotherm Study

The maximum equilibrium adsorption capacity was determined over the concentration range 0–200 mg L^−1^, recorded at intervals of 20 mg L^−1^. It can be observed that the adsorption capacity of CXTi increased with the increase in initial concentration of MB. A rapid increase in adsorption capacity was observed at low concentration, suggesting that CXTi has abundant active sites. With increasing MB concentration, the adsorption capacity reached a saturation plateau, indicating that the active sites were completely occupied by MB dye molecules. To understand the interactions between the adsorbent and adsorbate at equilibrium, adsorption isotherms obtained in this study were analysed according to the nonlinear form of Langmuir, Freundlich, and Sips isotherm models, and values of the associated model parameters were determined. The model showing the best fit to the experimental data, based on determination of the regression coefficient (R^2^), was selected for adsorption interpretation. The Langmuir isotherm model is relevant for the prediction of monolayer adsorption on energetically uniform homogenous adsorption sites, whereas the Freundlich isotherm model predicts multilayer adsorption [28]. Langmuir and Freundlich models are expressed in Equations (5) and (7), respectively.

(5) $$q_{e}=\frac{q_{L}K_{L}C_{e}}{1+C_{e}K_{L}}$$

where q_e_ (mg g^−1^) is the MB uptake at equilibrium, C_e_ (mg L^−1^) is the equilibrium concentration, q_L_ (mg g^−1^) is the amount of adsorbate at complete monolayer coverage, and K_L_ is the Langmuir constant related to the energy of adsorption, which can be used to determine the extent of adsorbate–adsorbent interaction. Furthermore, adsorption favourability can be determined by a dimensionless constant called the separation factor, R_L_, expressed in Equation (6):

(6) $$R_{L}=\frac{1}{\left(1+K_{L}C_{0}\right)}$$

where, C_0_ refers to the initial concentration of the adsorbate in (mg L^−1^), and K_L_ is Langmuir constant related to the adsorption capacity. If the value of R_L_ > 1, the adsorption is unfavourable, and favourable when 0 < R_L_ < 1. The Freundlich isotherm model is represented by:

(7) $$q_{e}=K_{F}C_{e}^{1/\mathrm{nF}}$$

where q_e_ (mg g^−1^) and C_e_ (mg L^−1^) are as defined in the Langmuir equation, adsorption affinity is related to the adsorption constant K_F_, and n_F_ indicates the magnitude of the adsorption driving force and is used to evaluate the adsorption favourability. When 1/n_F_ is greater than 0 (0 < 1/n_F_ < 1), adsorption is favourable; whereas, when 1/n_F_ is greater than 1, the adsorption process is unfavourable. Moreover, the adsorption intensity or surface heterogeneity and the energy distribution, as well as the adsorbate site heterogeneity, is indicated by 1/n_F_. A value of n_F_ between 2 and 10 represents good adsorption, indicating a high adsorption capacity, while values between 1 and 2 indicate moderate adsorption capacity, and values less than 1 indicate a small adsorption capacity. 

Sips, a three-parameter model, is a combination of Langmuir and Freundlich adsorption isotherm models. It is stated that the Sips model predicts characteristics of the Langmuir isotherm at higher concentrations, while predicting Freundlich isotherm behaviour at lower concentrations [29]. The Sips model is expressed in Equation (8):

(8) $$q_{e}=\frac{q_{s}K_{s}C_{e}^{n_{s}}}{1+K_{s}C_{e}^{n_{s}}}$$

where q_e_ (mg g^−1^) and C_e_ (mg L^−1^) are as defined in the Langmuir and Freundlich equations, 𝐾_𝑠_ is the Sips isotherm model constant (L g^−1^), and n_s_ is the Sips isotherm exponent, which is related to the heterogeneity factor that represents the deviation of the linearity of adsorption. The heterogeneity of adsorbents in the equation is illustrated by 1/n_s_; if 1/n_s_ < 1, the adsorbent surface is heterogeneous, and if 1/n_s_ ~ 1, the surface can be described as homogenous [29].

**Figure 9:**
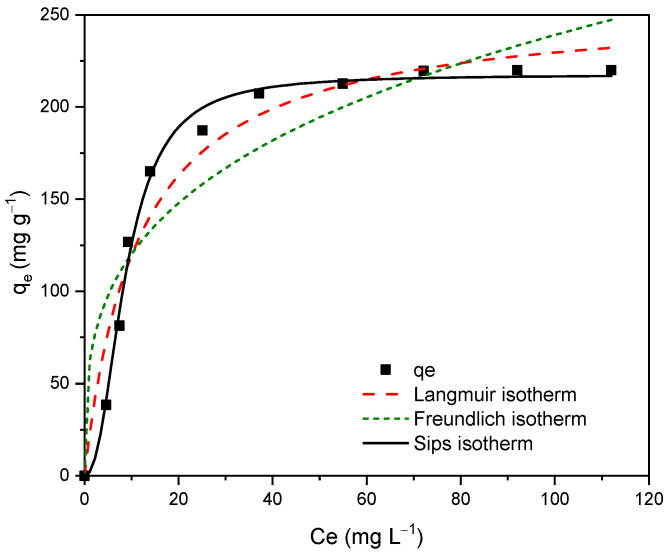
Adsorption isotherm fittings for Langmuir, Freundlich and Sips isotherm models for MB adsorption on CXTi synthesised in this study.

The fittings of the Langmuir, Freundlich and Sips isotherm models to adsorption data obtained for MB on CXTi are presented in Figure 9, and the calculated parameters are shown in Table 2. The fitting results based on regression coefficient values indicate that the Sips isotherm model exhibits the best fit to the experimentally obtained adsorption equilibrium data. The maximum adsorption capacity obtained from the Sips model (q_s_ = 217 mg g^−1^) was closest to the experimentally determined value (q_e_ = 218 mg g^−1^). The heterogeneity factor, n_s_, is greater than 1; therefore, the suggestion is that the adsorption surface is heterogeneous. The evaluated parameters from the Freundlich model also validate the heterogeneous nature of the sorbent, since the value of n_F_ is greater than 1. Additionally, the separation factor, R_L_ is less than 1, which indicates favourable adsorption. The isotherm models fit most appropriately in the order Sips > Langmuir > Freundlich. 

### 2.4. Photocatalytic Study

**Figure 10:**
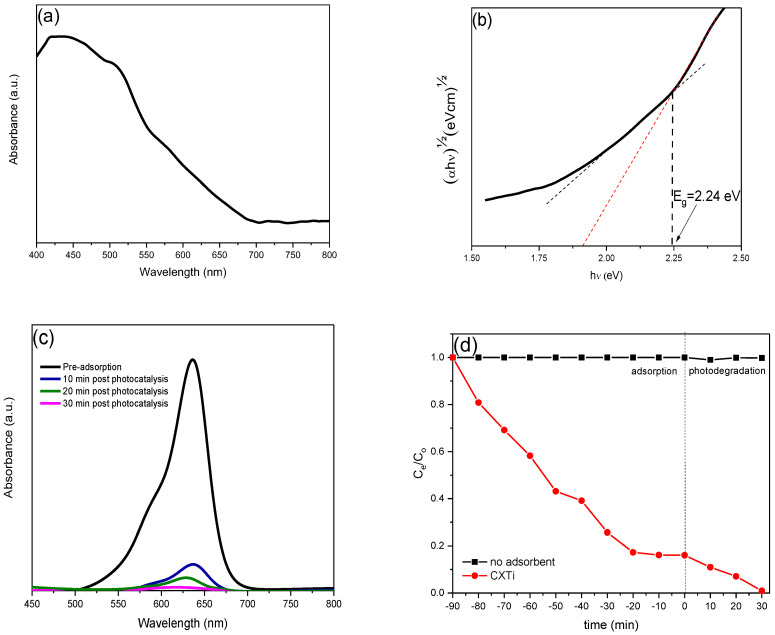
(**a**) UV-Vis absorption spectrum of CXTi synthesised in this study, (**b**) Tauc plot of CXTi, (**c**) post adsorption photocatalytic performance of CXTi under visible light irradiation and (**d**) kinetics of combined adsorption and photodegradation of MB on CXTi.

**Scheme 1:**
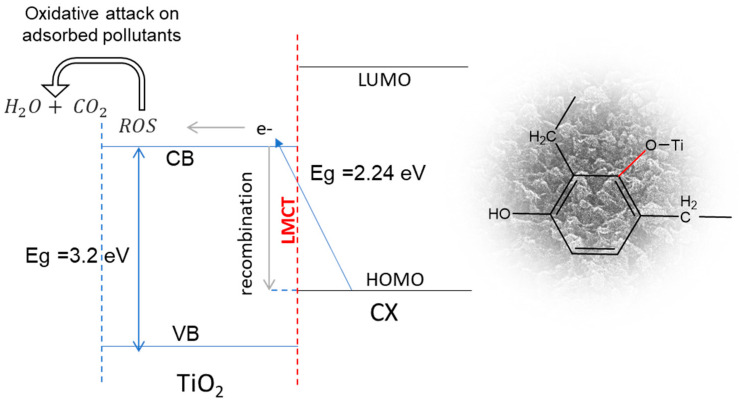
Proposed photocatalytic mechanism of CXTi under visible light irradiation.

As reflected in Figure 10a, absorption extends broadly to the visible region of the electromagnetic spectrum, with maximum absorption observed at 509 nm. The corresponding band gap calculated through the Tauc method was 2.24 eV [30]; meaning that CXTi can absorb and activate under visible light irradiation. This point was proven by performing further experiments post adsorption to observe decolourisation of MB under visible light. Figure 10c validates the reduction of MB, with a flattened curve observed after 30 min of exposure to light irradiation. Consequent reduction in MB concentration by combined adsorption–photocatalysis is shown in Figure 10d. It was observed that the MB removal capacity of CXTi increased from ~85 to 99% upon light irradiation. As can be seen in Table 3, these results are more efficient than those of similar systems. The photocatalytic activity in this work arises from the synergy of CX and TiO_2_. The chemical linkage between CX and TiO_2_ forms a surface complex via a hydroxyl group, where a single oxygen atom separates the phenolic ligand from the TiO_2_ surface [16]. This binding phenomenon is similar to that previously described for aromatic compounds with phenolic hydroxyl groups, which can chemically bind with functional groups of TiO_2_ [31]. Binding through the hydroxyl group enables strong coupling due to ligand–metal charge transfer (LMCT). The formation of LMCT complexes modifies the overall electronic structure and creates a new absorption band in the visible light [31,32], as illustrated in Scheme 1. The visible light photocatalytic activity occurs when light falls on the surface of CXTi, resulting in generation of photoexcited charge carriers (electron and hole pairs). These charge carriers transfer from the highest occupied molecular orbital (HOMO) of the CX to the conduction band of TiO_2_, similar to other chemically combined carbon/TiO_2_ systems [33]. Charge carriers successfully transferred to the surface of the photocatalyst take part in redox reactions and produce reactive oxide species (ROS), including hydroxy radicals (^●^OH) and superoxide radicals (O_2_^●−^) that can efficiently decompose adsorbed MB species [23,34]. 

**Figure 11:**
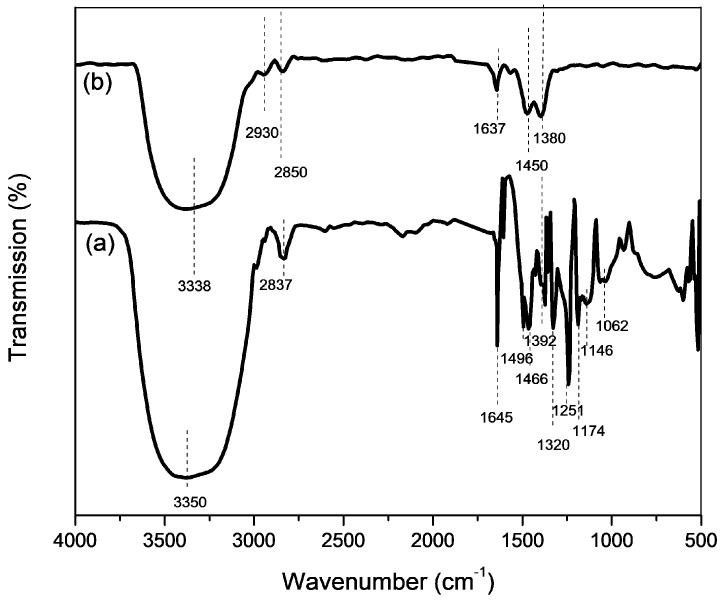
FTIR of (a) pure MB (b) MB after photodegradation.

The change in structure of MB after photodegradation was confirmed by recording FTIR spectra shown in Figure 11 before (a) and after (b) photocatalytic activity. The differences in both the spectra clearly validate the change in structure of the MB dye molecule post treatment, and are in agreement with the previous studies [39,40]. The main functional moiety associated with MB was detected at ~2900 cm^−1^ for methylene asymmetric stretching (C-H). The other functional moieties are observed for overlapped OH and NH at 3350 cm^−1^, CH=N at 1645 cm^−1^, C=C side rings in the range of 1500 to 1400 cm^−1^, CH_3_ or CH_2_ stretching in the range of 1400 to 1300 cm^−1^, C-N stretching absorption peaks at 1252 cm^−1^, C-H at 1176 cm^−1^, C-N at 1146 and C-S-C at 1060 cm^−1^, and C-H out plane bending observed in the range of 800 cm^−1^. After photodegradation of MB by ROS produced by CXTi under visible light, most of the characteristic absorption peaks linked with MB weakened or disappeared, which suggests the breakdown of the MB molecule at the interface of the CXTi. The main decomposed peaks were observed at 3338 and 1637 cm^−1^ for H-OH, 1470 for N-H and 2930 and 2850 cm^−1^ for C-H, which suggests successful degradation of MB by CXTi under visible light irradiation. 

**Figure 12:**
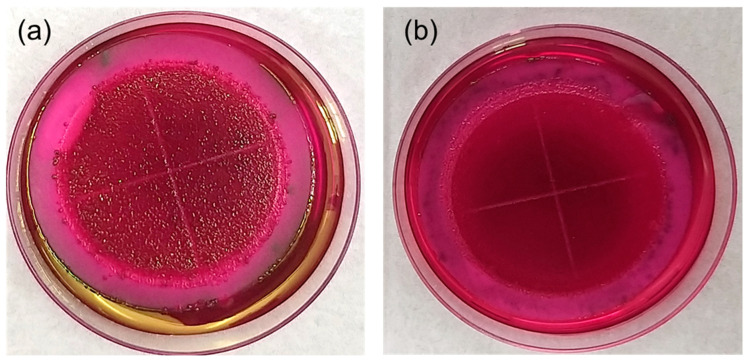
Pictures of petri plates for detection of total coliforms following 24 h incubation at 35 °C (**a**) before water treatment and (**b**) after water treatment with CXTi. The colonies that were dark red, mucoidal and had dark centres were counted. Dark centres that produce metallic sheen are considered to be total coliform colonies. Metallic green colonies indicate the presence of *E. coli* in a water sample.

**Figure 13:**
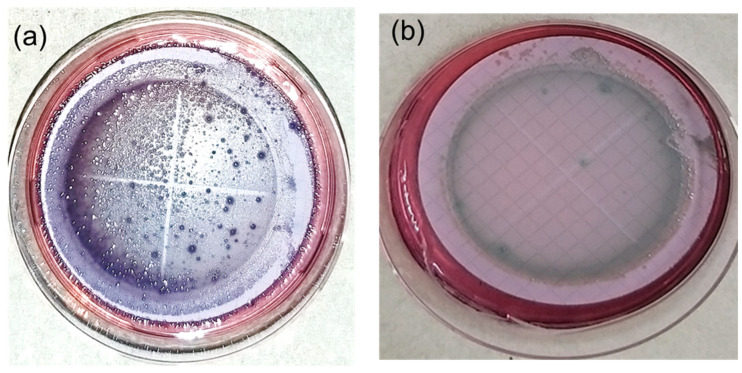
Pictures of petri plates for detection of faecal coliforms following 24 h incubation at 44.5 °C (**a**) before water treatment and (**b**) after water treatment with CXTi. Only blue colonies are counted, which are considered to be faecal coliform colonies. *E. coli* form dark blue flat colonies.

Photogenerated ROS have been reported to kill bacteria by attacking the cell wall, leading to cell rupture, reduction in growth and ultimately cell death [34]. It has been reported that the usual first target of ROS is the cell wall; e.g., in *E. coli*, the cell wall is composed of lipopolysaccharide, peptidoglycan and phosphatidyl-ethanolamine, which have been reported to be affected by ROS [41]. Secondly, the rupture of the cell membrane occurs, leading to leakage of cellular matter and the ultimate breakdown of the cell [42]. Thirdly, cell lysis progresses by inhibiting the respiratory chain, followed by damage to DNA [43]. The antimicrobial performance of CXTi was studied by conducting tests using a membrane filtration procedure according to a standard method (9222 membrane filter technique for members of the coliform group [44]). The performance was tested against the reduction in total and faecal coliform bacteria and *E. coli*. After treating contaminated water with CXTi, the calculation of bacteria was performed by counting the number of colonies, colony forming units (CFU per 100 mL) and developed post incubation colonies grown on the grid after 24 h. Pictures of petri plates before (control) and after (surviving bacteria) treatment are shown in Figure 12 and Figure 13, and numerical data is presented in Table 4. The reduction in colonies signifies complete eradication of almost all surviving bacteria. Remarkable antimicrobial activity is attributed to the production of a sufficient amount of ROS to attack the bacterial cell wall for complete destruction. 

## 3. Materials and Method

### 3.1. Synthesis of CXTi

CX and TiO_2_ were combined using a sol–gel process. 5.43 g of resorcinol (SigmaAldrich, ReagentPlus, 99%, Poole, UK) was completely dissolved in 50 mL of deionised water. 0.02 g of catalyst, sodium carbonate (Na_2_CO_3_, Sigma-Aldrich, anhydrous, ≥99.5%) and 2.96 g of formaldehyde (37 wt%) were added to the resorcinol solution under continuous stirring, at room temperature. The pH recorded at this point was 7.4. A titania sol* was obtained using a conventional method described elsewhere [45], and added dropwise to the system. The integrated system was stirred at 23 °C for 2 h, after which the sol mixture was aged at 85 °C for 72 h. Aging was followed by solvent exchange through immersion of wet monolithic CXTi in acetone. After 72 h, CX Ti was dried in a vacuum oven (Townson and Mercer 1425 Digital Vacuum Oven) at 110 °C for 48 h, and the ultimate CXTi was obtained with 30 wt% TiO_2_ (theoretical percentage).

*Briefly, 3.6 g of precursor, titanium isopropoxide (TTIP) (98+%, ACROS Organics™, Geel, Belgium), was mixed with ethanol, followed by dropwise addition of HCl and water solution in the following molar ratio: 1 TTIP:10 EtOH:0.3 HCl:0.1 H_2_O. The mixture was stirred at room temperature for the hydrolysis reaction to occur, and a homogenous solution was obtained after 2 h of agitation. The anatase phase of TiO_2_ deposited was confirmed by X-ray diffraction spectrum [46,47,48] (displayed in Figure S2, Supplementary Materials).

### 3.2. Structural Characterisation

Morphology of the materials was studied using micrograph images obtained via field emission electron scanning microscopy (FESEM) TESCAN-MIRA. The chemical linkages were investigated through Fourier Transform Infrared (FTIR) Spectroscopy (MB3000 series, scanned in the range 4000–400 nm, at an interval 4 cm^−1^, over 16 scans). Surface area was studied by obtaining N_2_ adsorption isotherms at −196 °C (Micromeritics ASAP 2420) and using BET analysis; pore size was estimated using BJH theory [20]. Absorption vs. wavelength spectra were obtained using UV-Vis Spectrophotometry (Varian Cary 5000 UV-Vis NIR Spectrophotometer Hellma Analytics).

### 3.3. Adsorption and Photocatalytic Performance 

The adsorption experiments were conducted by adding 0.01 g of CXTi to 25 mL of prepared concentrations of MB solution in the range 20–200 mg L^−1^. The pH of the solutions was adjusted if required using 1M HCl and 1M NaOH. The adsorption equilibria were established by agitating the systems using an orbital shaker (VWR 3500 Analog Orbital Shaker unit), set to 125 rpm at 23 °C, in the dark. After a given time, the solution was centrifuged for 15 min and the supernatant was collected for measurement. The concentration of the treated solution was determined using UV-Vis spectrophotometry (Varian Cary 5000 UV-Vis NIR Spectrophotometer Hellma Analytics). Likewise, post adsorption, the concentration after photocatalytic treatment was determined at given time intervals of exposure to visible light (irradiance 111 W m^−2^).

The equilibrium adsorption capacity, q_e_ (mg g^−1^), was calculated using

(9) $$q_{e}=\frac{\left(C_{o}-C_{e}\right).V\left(l\right)}{W}$$

The corresponding percentage removal of MB was calculated by

(10) $$\mathrm{Removal}\;\%=\frac{C_{o}-C_{e}}{C_{o}}\times 100\%$$

where C_o_ and C_e_ are the initial MB and final concentration, respectively. W is the weight (g) of the adsorbent and V is the volume (L) of MB solution. 

The effect of contact time was determined using aliquots of MB solution (25 mL, 100 mg L^−1^) and 0.01 g adsorbent gel, added into flasks and agitated for contact times in the range 5 min to 4 h. The samples were prepared and treated as above, and the amount of adsorption was calculated using Equation (11).

(11) $$q_{t}=\frac{\left(C_{o}-C_{t}\right).V}{W}$$

where C_o_ and C_t_ are the initial MB and equilibrium concentration at a given time, respectively, V is the volume of solution (L), and W is the mass of adsorbent sample in (g). Equilibrium concentration was determined by plotting $q_{t}$ versus time of aliquots collected, at different time intervals.

### 3.4. Antimicrobial Performance

A stock solution of lab-cultivated bacteria was prepared with volume ratio 1:100. For the detection of bacteria, mEndo and mFC agars were prepared, and a membrane filtration procedure (MF) was performed according to 9222 standard methods for the examination of water and wastewater using the membrane filter (MF) technique for members of the coliform group [44]. 

Briefly, 250 mL transparent sterile water bottles were filled with 200 mL of contaminated water. According to standard microbiological examination (9000), the suggested sample volume to be filtered using membrane filtration for coliform or *E. coli* testing in drinking water is 100 mL. Therefore, 100 mL of each treated sample was measured twice, once for detection of *E. coli*, and the remaining 100 mL for detection of faecal coliform. The bottles were labelled with the respective sample codes and 0.1 g of CXTi was added to the contents of the bottle. After preparation, sample bottles were placed on an orbital shaker (VRN 360 Gemmy, Taipei, Taiwan) at 200 rpm for 90 min, to establish equilibrium. The bottles were then exposed to visible light. CXTi was filtered out and the membrane filtration procedure was carried out. 100 mL of each treated water sample was filtered twice using microfiltered paper, which was placed on mEndo and mFC agar petri plates. These plates were incubated for 24 h, at 35 °C for mEndo and at 44 °C for mFC agar plates. mEndo (pink plates) form a dark red, mucoid or dark centre without metallic sheen. *E. coli* will form colonies with a metallic sheen. mFC (blue plates) are for the detection of faecal coliform. Faecal coliforms form blue colonies on this medium, and *E. coli* will form flat dark blue colonies. The grown bacterial colonies were counted according to the standard counting procedure. The cell density of the original sample was calculated and compared with the cell density of water treated with CXTi. The counts were reported as coliform colony units (CFU/100 mL). 

## 4. Conclusions

**Figure 14:**
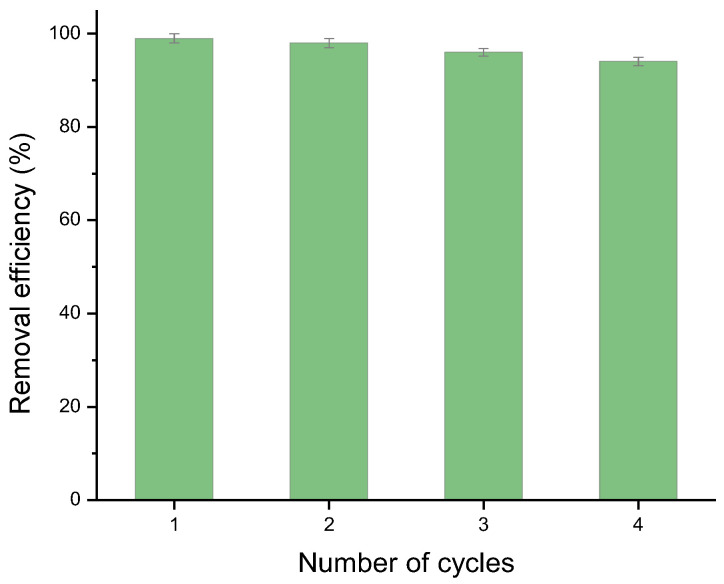
CXTi reusability after testing against degradation of MB dye (C_0_ = 100 mg L^−1^, T = 23 °C, dose = 0.01 g mL^−1^, total treatment time 120 min).

The CXTi adsorbent–photocatalyst was successfully synthesised within this study, using a sol–gel method to combine a resorcinol–formaldehyde xerogel (CX) with titanium dioxide (TiO_2_). As expected, the integration of TiO_2_ into the carbon xerogel material modified the electronic structure of TiO_2_, which enabled visible light response, and the CXTi sample demonstrated efficient adsorbent–photocatalyst behaviour for the removal of MB under given conditions. The formation of a heterojunction between TiO_2_ and the CX material was confirmed using spectroscopic methods. The kinetics of MB adsorption revealed that agitation for 90 min was sufficient to attain equilibrium, and the kinetic profiles better fitted a pseudo second order model, indicating chemical processes are involved within MB removal. The equilibrium adsorption data were best described by the Sips model, suggesting heterogeneity of the sample surface, which is fully in line with the mixed material composite produce here. Additionally, the mesoporous carbon phase provides a high surface area for optimised adsorption, and photocatalysis is enabled by the inclusion of TiO_2_, resulting in the complete eradication of microbes under given conditions. The excellent adsorption–photodegradation abilities exhibited by CXTi, achieved through the synergistic effects of combining CX and titania, present an economically viable option for water treatment, as they can be effectively recovered and reused. Recycling efficiency tests, against the reduction in MB, demonstrated a minimal loss (~5%) in dye degradation efficiency by the fourth repeated cycle (Figure 14), suggesting good reusability of over 90% efficiency with repeated use. Thus, CXTi is a promising candidate for efficient removal of a wide range of synthetic azo dyes, as well as microbes, from industrial effluents.

## Figures and Tables

**Table 1:** Kinetic constants for MB adsorption by CXTi; q_exp_ is the experimental amount adsorbed at equilibrium.

	50 mg L^−1^	100 mg L^−1^	150 mg L^−1^	200 mg L^−1^
**q_exp_**	113	217	220	221
**Pseudo first order**				
**q_t_ mg g^−1^**	112	211	216	217
**k_1_ min^−1^**	0.104	0.119	0.114	0.177
**R^2^**	0.963	0.959	0.947	0.975
**Pseudo second order**				
**q_t_ mg g^−1^**	120	225	230	229
**k_2_ (10^−3^) g mg^−1^ min^−1^**	1.40	0.875	0.839	1.43
**R^2^**	0.993	0.993	0.990	0.999
**Elovich**				
**R^2^**	0.976	0.977	0.979	0.979

**Table 2:** Parameters for MB adsorption at equilibrium computed from Langmuir, Freundlich and Sips isotherm model equations.

Isotherm Model	Parameters	
Experimental	q_e_ (mg g^−1^)	218
Langmuir	q_L_ (mg g^−1^)	255
	K_L_ (L mg^−1^)	0.087
	R_L_	0.103
	R^2^	0.951
Freundlich	K_F_ mg g^−1^ (L mg^−1^)^1/n^	60.5
	n_F_	3.35
	R^2^	0.861
Sips	q_s_ (mg g^−1^)	217
	K_s_ (L mg^−1^)	0.007
	n_s_	2.28
	R^2^	0.992

**Table 3:** Characteristics and degradation performance of comparable systems.

Material	Procedure	Inorganic Content	Surface Area m^2^ g^−1^	Band gap (eV)	Pollutant	Removal Efficiency	Ref.
Magnet photocatalyst based on Carbon Xerogels/TiO_2_	Photoinactivation under UV irradiation	Fe_3_O_4_/SiO_2_/N-CXTi = 70% Fe_3_O_4_/N-CXTi = 67%	Fe_3_O_4_/SiO_2_/N-CXTi = 388 Fe_3_O_4_/N-CXTi = 212	Fe_3_O_4_/SiO_2_/N-CXTi = 3.22 Fe_3_O_4_/SiO_2_/N-CXTi = 3.10	Acetaminophen	Fe_3_O_4_/SiO_2_/N-CXTi = 98% Fe_3_O_4_/SiO_2_/N-CXTi = 85%	[35]
Carbon/TiO_2_ gels	Adsorption–Photodegradation under UV–Vis irradiation	10%	588	2.97	Methylene blue, microbes	74%	[17]
Carbon Xerogel/Ti	Simultaneous adsorption–photodegradation	38%	650	2.68	Acetaminophen	96%	[36]
Carbon Xerogel/TiO_2_ composite	Adsorption–photodegradation	33%	401	2.60	Orange G	70%	[37]
TiO_2_/Carbon Aerogel composite	Photoinactivation under UV irradiation	85%	124	-	Dimethyl phthalate (DMP)	83%	[38]
Carbon Xerogel/Titania	Adsorption–photodegradation	30%	384	2.24	Methylene blue, Indicator microorganisms	99%	This work

**Table 4:** Antimicrobial results after performing tests against reduction in total and faecal coliform and *E. coli* in control conditions and treatment using CXTi synthesised in this study.

	Total Coliform	Faecal Coliform	*E. coli*
**Control**	210	180	176
**Post Treatment with CXTi**	<1	<1	<1

## Data Availability

Not applicable.

## Supplementary Materials

The following supporting information can be downloaded at: https://www.mdpi.com/article/10.3390/molecules27238483/s1, Table S1: Parameters calculated from piecewise linear fittings to Intra-particle diffusion model, corresponding to Figure 7. Figure S1: X-ray diffraction (XRD) spectrum of Carbon Xerogel (CX) indicating two broad diffraction peaks of CX at 2θ = 24° and 2θ = 44°, represented by highlighted region in light grey. These findings are similar to previously described XRD pattern obtained for CX derived from resorcinol-formaldehyde [46,47]. Figure S2: X-ray diffraction spectrum of Carbon Xerogel/TiO_2_ (CXTi) indicating the presence of anatase phase at 2θ = 25°, represented by highlighted region light grey. These findings are similar to previously described carbon/titania systems [48].
