# Supplementary material for: Integrated Adsorption–Photodegradation of Organic Pollutants by Carbon Xerogel/Titania Composites
Source: Molecules. 2022 Dec 2;27(23):8483. doi: 10.3390/molecules27238483 (PMC9735923; doi:10.3390/molecules27238483)
Supplement: Supplementary file 1 [file molecules-27-08483-s001.zip › molecules-2038857-supplementary.pdf]

Integrated Adsorption-Photodegradation of Organic Pollutants by Combined Photoactive Carbon Xerogel/Titania adsorbent

Anam Safri [1], Ashleigh Jane Fletcher [1*], Ramsha Safri[2], and Hifza Rasheed[3]

[1] Department of Chemical and Process Engineering, University of Strathclyde, Glasgow G1 1XJ, UK
[2] The Shaheed Zulfiqar Ali Bhutto Medical University Islamabad, Pakistan

[3] Pakistan Council of Research in Water Resources (PCRWR), Islamabad, Pakistan

* Correspondence: ashleigh.fletcher@strath.ac.uk; Tel.: +44-141-5482-431

Supplementary information

Table S1. Parameters calculated from piecewise linear fittings to Intra-particle diffusion model, corresponding to Figure 7.

**Concentration mg L^{-1}**	**Stage 1**	**Stage 2**	**Stage 3**
**50**			
**C**	3.93	75.9	104
**k_{ip}1**	21.3	4.36	0.872
**R^2**	0.950	0.978	0.971
**100**			
**Intercept**	4.22	145	207
**k_{ip} 2**	45.9	7.36	0.953
**R^2**	0.943	0.791	0.956
**150**			
**C**	10.7	139	210
**k_p 3**	42.0	9.89	1.08
**R^2**	0.909	0.984	0.996
**200**			
**C**	10.4	185	218
**k_{ip} 4**	50.0	3.77	0.525
**R^2**	0.938	0.696	0.784

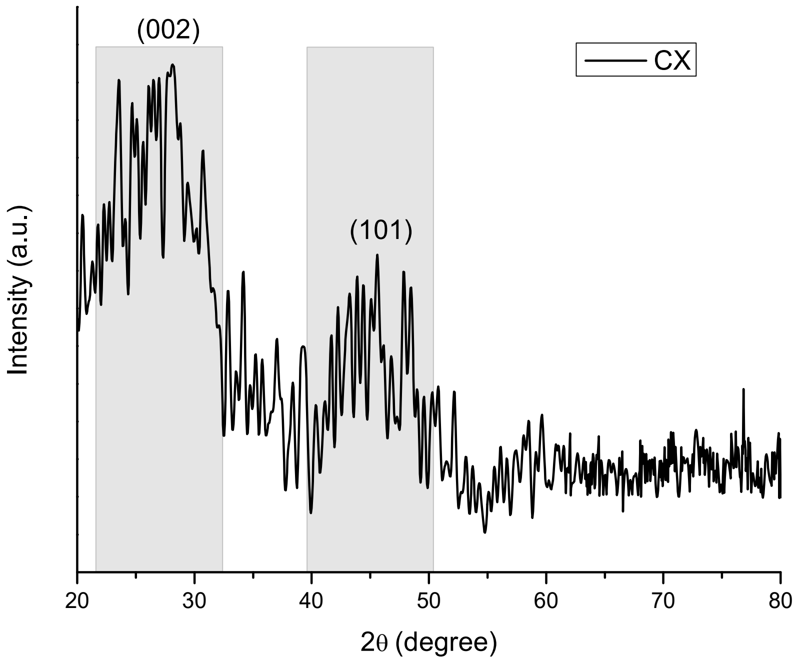

Figure S1. X-ray diffraction (XRD) spectrum of Carbon Xerogel (CX) indicating two broad diffraction peaks of CX at 2θ = 24° and 2θ = 44°, represented by highlighted region in light grey. These findings are similar to previously described XRD pattern obtained for CX derived from resorcinol-formaldehyde [46,47].

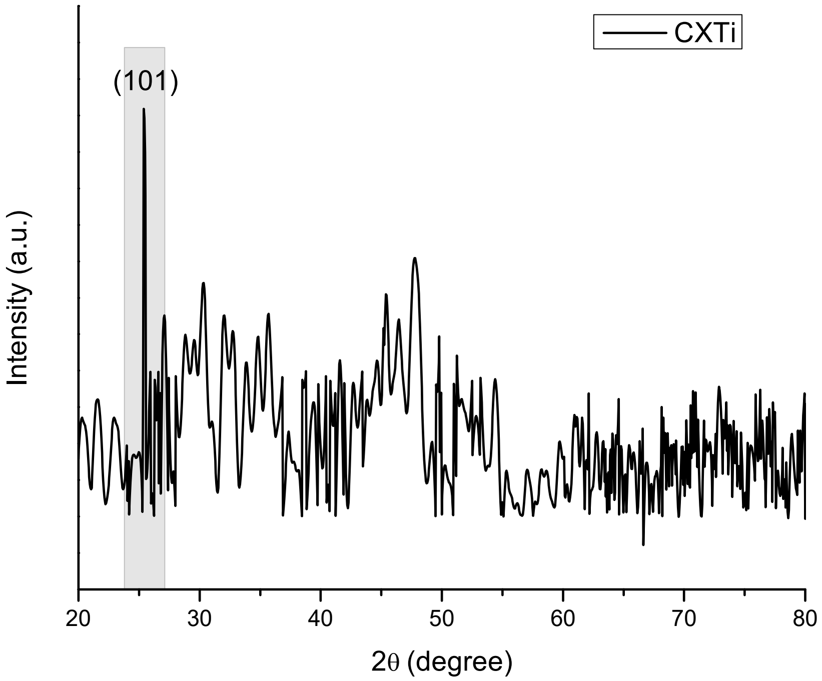

Figure S2. X-ray diffraction spectrum of Carbon Xerogel/TiO_2 (CXTi) indicating the presence of anatase phase at 2θ = 25°, represented by highlighted region light grey. These findings are similar to previously described carbon/titania systems [48].
